# Reduction of CO_2_ Emissions in Ceramic Production from Clay Raw Materials Containing Carbonates

**DOI:** 10.3390/ma19132851

**Published:** 2026-07-03

**Authors:** Wojciech Wons, Karol Rzepa, Agnieszka Wojteczko

**Affiliations:** 1 Department of Building Materials Technology, Faculty of Materials Science and Ceramics, AGH University of Krakow, A. Mickiewicza Av. 30, 30-059 Krakow, Poland; krzepa@agh.edu.pl; 2 Department of Ceramics and Refractories, Faculty of Materials Science and Ceramics, AGH University of Krakow, A. Mickiewicza Av. 30, 30-059 Krakow, Poland; agdudek@agh.edu.pl

**Keywords:** clays, decarbonization, CO_2_ emission, building ceramics, thermal properties, sintering

## Abstract

The production of building ceramics is an energy-intensive part of the industry, causing high CO_2_ emission per production volume. In addition to the combustion of fossil fuels, CO_2_ is emitted as a byproduct of calcium carbonate decomposition, a compound present in clay raw materials. In this paper, a method for reducing emissions by lowering the firing temperature of ceramics, thereby preventing the complete decarbonation of carbonate minerals, is presented. Thermal research has shown that lowering the firing temperature to 750 °C resulted in a 55% calcium carbonate decomposition and a reduction in CO_2_ emissions by over 30 kg for every ton of clay used. At this temperature, sintering shrinkage mechanisms were not observed, which resulted in a reduction in the strength of the materials by almost 25% compared to samples fired at 900 °C. An attempt was made to compensate for the negative effects of lowering the firing temperature by adding ground glass cullet, which brought only partially positive results: an increase in flexural strength, but no change in compressive strength. Microscopic observations and phase composition studies indicate that lowering the firing temperature causes changes in the proportions of calcium compounds: increased amounts of calcite, and decreased amounts of silicates and calcium aluminosilicates.

## 1. Introduction

Ceramics are a set of inorganic and non-metallic materials obtained by sintering powders [1]. Although ceramic is the oldest material produced by humans [2], its importance, use and production volume are constantly increasing. In 2023, the estimated value of the global ceramics market was almost USD 248 billion [3]. In terms of production value, the leading sectors of the ceramics industry are building ceramics (bricks, blocks and roof tiles), wall and floor tiles, and refractory materials [4,5]. These industrial sectors are energy-intensive, with energy-related costs at approximately 30% [6,7,8]. Most of the necessary energy is obtained from the combustion of fossil fuels; hence, the emission of exhaust gases, including greenhouse gases (such as CO_2_ and water vapor) is an inherent aspect of ceramic material production. According to the literature [9], in 2019, the production of bricks and roof tiles alone was responsible for 2.7% of global CO_2_ emissions. According to another estimate from 2007 [5], the total global emissions of the ceramic industry are 400 Mt CO_2_ per year. There are many studies on reducing CO_2_ emissions in other high-emission industries such as the cement [10,11] and concrete [12] industries, energy production [13,14] and steel [15] and copper [16] production. Although the ceramics industry’s emissions are significant, there are only few studies on the subject. Research by Furszyfer Del Rio and others [5] appeared in 2021, comprehensively addressing methods for reducing CO_2_ emissions in this part of the industry. They found that most CO_2_ emissions result from the combustion of fuels during the firing and drying processes. For example, in ceramic factories in the EU, fuel combustion is responsible for 66% of CO_2_ emissions, and, in the case of ceramic tile production, it can reach up to 90% [17,18,19]. In addition to fuels, the final balance of CO_2_ emissions includes emissions from electricity (18% share in the EU) and so-called process emissions (16% share in the EU) [20]. The ceramics industry’s emissions are steadily decreasing thanks to the introduction of new technical solutions. It is worth mentioning the most important ones:

1. Replacing high-emission fuels, such as coal, with low-emission fuels, such as gas [21].
2. Application of new devices: roller kilns for firing tiles, and tunnel kilns and dryers in the production of building ceramics [22].
3. Maximum heat recovery from the firing process for the drying of raw materials, and the use of cogeneration systems [22,23,24].
4. Changes in the thermal–time curves of the firing and drying processes.

CO_2_ emissions related to electricity are an indirect form of emissions. Its share in the ceramics industry’s total emissions depends primarily on the specific electricity generation methods used by a given country or power plant. Energy obtained from fossil fuel combustion increases the emission intensity of the energy industry. On the other side of the spectrum are energy sources based on renewable energy and nuclear power. Of course, power plants can also internally reduce their share of CO_2_ emissions from electricity through logistical measures, such as replacing devices with more energy-efficient ones or optimizing their operation.

The last sources of CO_2_ emissions are process emissions, related mainly to the specificity (phase composition) of the raw materials used; hence, it can also be described as raw material emissions. Part of the components contained in ceramic raw materials undergo reactions during thermal treatment with CO_2_ as a byproduct. These components include calcium, magnesium, iron carbonates and organic compounds. Raw material-related emissions are the most difficult to reduce because they are associated with the specific raw material deposits from which ceramic products are made. The presence of carbonates in ceramic raw materials is particularly unfavorable, as their thermal treatment not only causes CO_2_ emission in the decarbonation process, but also significantly increases the energy demand, as the decarbonation reaction requires a significant amount of heat: 177.9 kJ/mol [25]. Currently, the only practical way to reduce raw material emissions is to reduce the share of raw materials containing carbonates in the ceramic mass. The aim of this paper is to partially reduce process emissions by lowering the ceramic firing temperature below the end of the decomposition reaction of calcium carbonate contained in the clay raw material. Lowering the firing temperature results in less intensive sintering, the process which is responsible for the consolidation of grains and reducing the porosity of the material. It can therefore be expected that ceramic materials obtained after lowering the sintering temperature will be characterized by lower strength and higher porosity. According to the thesis of the paper, these effects can be compensated by the addition of ground glass cullet. This type of solution is not a novelty in ceramics. Soda-lime glasses are known for their fluxing properties, which is why they are used as a replacement for natural sodium fluxes, primarily in the production of ceramic tiles [26,27]. Glass cullet can also be used as a flux in traditional porous masonry ceramics [28,29,30]. There are also other known uses of cullet in the ceramics industry, including the production of ceramic engobes [31], magnetic ceramics [32] and refractory bricks in synergistic mixtures with fly ash [33].

## 2. Materials and Methods

### 2.1. Conception

The thesis of this study is that it is possible to reduce the ceramics sintering temperature in order to partially or completely limit the decarbonation (thermal dissociation) of calcium carbonate contained in the clay raw material, without significantly worsening the properties of ceramic materials. This is possible by using finely ground float glass cullet. The first research step was to determine the firing temperature range that would meet the assumption of incomplete decarbonation. For this purpose, differential thermal analysis (DTA) combined with thermogravimetry (TG) with gas analysis (EGA) was used. The decarbonization range was precisely determined based on derivative thermogravimetry (DTG) and the CO_2_ emission range. Then, the shrinkage sintering intervals of the ceramic mixtures were determined using a hot-stage microscopy (HSM).

Another important study, crucial for material durability, was the analysis of the microstructural effects resulting from lowering the ceramic material firing temperature. This involved a comparative analysis of ceramics sintered at various temperatures, ranging from 700 °C to 900 °C. As part of this research stage, phase composition tests were performed (XRD, DTA/TG and free CaO content using the glycol method), as well as microstructural tests of SEM/EDS sections combined with the distribution of elements (mapping).

The final research stage involved technological testing, which allowed for the assessment of the applied modification’s impact on the ceramic products performance parameters. For research purposes, three raw material mixtures were composed: pure Miocene clay and two mixtures with varying amounts of glass cullet as a flux. The compositions and designations of the mixtures are provided in Table 1.

The last row of Table 1 shows a sample of pure G10 glass cullet, which was not subject to technological testing, but was characterized. Pure clay samples were fired at four different temperatures (700 °C, 750 °C, 800 °C and 900 °C), while clay–cullet mixtures were fired at only two temperatures—700 °C and 750 °C—at which the thermal decomposition of carbonates, according to the thesis, is incomplete.

### 2.2. Materials

The basic raw material used was a clay material from the Miocene marine period, which has industrial applications in the production of masonry ceramics. Due to the aquatic conditions of this raw material’s geological formation (sedimentation combined with bioaccumulation), it contains dispersed calcite. Ground glass cullet was used as an ingredient to lower the firing temperature. Cullet is a waste fraction from soda-lime glass, which cannot be reused for glass production due to its impurities and fine grain size [34]. The flux properties of this waste are mainly determined by the high Na_2_O content (15.58%). The chemical composition analysis of clay raw material and glass cullet are presented in Table 2. The clay raw material contains approximately 6.8% CaO, which occurs primarily in the form of calcite. Assuming that CaO occurs exclusively in the form of calcium carbonate, the molar masses (M_CaO_ = 56, M_CaCO3_ = 100) indicate that the share of this phase in the clay raw material is approximately 12.2%.

**Figure 1:**
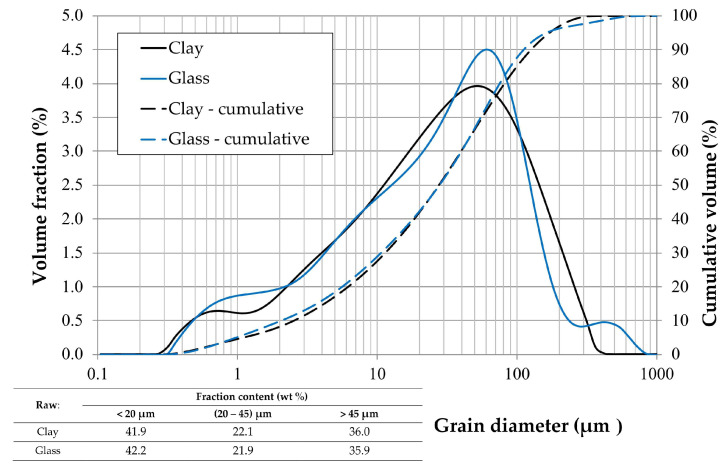
Grain size distribution of raw materials.

The grain size distribution of the clay raw material and ground glass cullet is presented in Figure 1. The grain compositions of both raw materials are similar.

### 2.3. Methods

The chemical composition analysis of clay and glass cullet were conducted using a PANalytical Axios mAX 4 kW WD XRF fluorescence spectrometer equipped with a Rhodium source (Almelo, The Netherlands).

Grain size distribution was determined by the laser method using a Malvern Mastersizer 2000 apparatus produced by Malvern Panalytical (Malvern, Worcestershire, UK) in water as a dispersion medium.

Thermal differential analysis coupled with the thermogravimetry DTA/TG method with gas analysis measurements were performed in a STA 449F3 Jupiter Netzsch apparatus (Selb, Germany). The rate of heating was 15 °C/min. The measurements were connected with gas analysis by EGA evolved gas analysis (QMS—quadruple mass spectrometer) and were initially carried out in an atmosphere of synthetic air a flow of 40 mL/min. Gas analysis was performed from the molar mass range from 1 to 70.

The phase composition analysis was performed for clay raw material and sinters from different temperatures. Phase composition was determined by X-ray diffraction (XRD) using a PANalytical X’Pert Pro MD diffractometer (Amsterdam, The Netherlands). The measurement was carried out in the range of deflection angles (2θ) from 5° to 60° for X-rays, the source of which was a copper anode lamp. A 0.05° step was applied at a counting rate of 1° of the angle 2θ per minute.

Another method that was used for examination of the behavior of materials during high-temperature processing was hot-stage microscopy. The essence of this method consists of the registration of the size and shape of the samples during thermal treatment. In a special manual press, cylindrical samples with dimensions d = h = 3 mm were prepared; samples of ceramic mixtures and clay were measured using a Hesse Instruments hot-stage microscope (HSM) (Osterode am Harz, Germany). The measurement was carried out at a constant heating rate of 10 °C/min up to a temperature of 1400 °C in an air atmosphere. During the measurement, so-called characteristic temperatures in HSM were determined:

- Beginning of sintering (first shrinkage during sintering);
- Maximum of sintering (maximum shrinkage during sintering without deformation);
- The softening point (first deformation of sample);
- Maximum of expansion (maximum area of sample, which is caused by pyroplastic expansion);
- The melting point (sample is hemispherical).

The surfaces of the sintered ceramic cross-sections were observed using a high-resolution Apreo 2S LowVac scanning electron microscope (Thermo Scientific, Brno, Czech Republic) equipped with a Schottky field emission gun. Observations were performed using a backscattered electron (BSE) detector, and elemental composition analysis was performed using an Octane Elect EDS detector (EDAX, Mahwah, NJ, USA). The accelerating voltage was 15 kV. Prior to observation, the ceramic sections were coated with a nanometric carbon layer to ensure electrical conductivity and charge dissipation. EDS maps were acquired using EDAX APEX Advanced (3.0.0601.0001) with a 2500× magnification level at a resolution of 1248 × 975 pixels. To estimate the composition of sinters phases, based on the received mapping, the Glueviz (1.21.1) Python library with the EDXIA (0.1.13) python package, and the GIMP (3.2.4) image editor and ImageJ (1.54t) were used.

The content of free CaO was determined using the ethylene glycol method [35]. The glycol method involves the selective extraction of free CaO from a powder sample using ethylene glycol heated to 70 °C ± 5 °C, which reacts with CaO to form a complex compound. The resulting compound is then titrated with a standard 0.1 M HCl solution in the presence of an indicator, allowing for precise calculation of the CaO content.

Ceramic samples were prepared in the following procedures. The clay raw material was dried until it reached a constant weight, and subsequently crushed to achieve a grain size smaller than 2 mm using a mechanical disintegrator. The glass cullet was ground for 30 min in a laboratory ball mill, with spherical steel grinding media with a diameter of 30–50 mm. The grain size distribution of the ground glass cullet is shown in Figure 1. Following the mixing of raw materials, water was added until a plastic mass was attained. A laboratory screw press with venting from VERDES was used to homogenize the plastic mass and form the samples. This type of press is a laboratory replica of a press that molds samples under factory conditions. This ensures that the samples are homogeneous and well-ventilated, ensuring repeatable results with minimal scatter. The samples were in the form of cuboids (bricks) of 20 mm × 30 mm × 60 mm, beams of 20 mm × 30 mm × 150 mm and cubes of 50 mm × 50 mm × 50 mm. The prepared samples were first dried in laboratory conditions, then in a laboratory dryer at the maximum temperature of 105 °C. Next, the dried samples were fired in a laboratory chamber furnace under the following conditions: temperature increased at a rate of 100 °C/h, held at 600 °C for 1 h, then held at the maximum temperature for 1 h, followed by inertial cooling. In the microstructural tests (Section 3.2), cuboids (bricks) were used—20 mm × 30 mm × 60 mm, fired at maximum temperatures ranging from 700 °C to 900 °C. The technological test parameters (Section 3.3) were determined in the following manner for the following sample types:

Firing shrinkage (S_F_) was determined on cuboid samples by measuring the spacing of the markers after drying—m_d_, and after firing—m_w_. The calculation was made based on the following equation:

(1) $$S_{F}=\frac{m_{w}-m_{d}}{m_{w}}\cdot 100\%$$

The bulk density d was calculated based on the following equation:

(2) $$d=\frac{w_{dry}}{a\cdot b\cdot h}$$

where w_dry_—weight of the dry sample after firing, a, b, h—cube sample dimensions. Water absorption WA was determined on cuboid samples by comparing the weight of water absorbed by the sample after 48 h of saturation w_wet_ to the weight of the dry sample w_dry_ and was calculated using the equation:

(3) $$WA=\frac{W_{wet}-w_{dry}}{w_{dry}}\cdot 100\%$$

Flexural strength was measured on beam samples by the three-point bending method using the universal test machine Cometech QC-508 (Taichung, Taiwan) with a press arm speed of 2 mm/min. The flexural strength R_flex_ was calculated using the equation:

(4) $$R_{flex}=\frac{1.5\cdot F_{flex}\cdot S}{a\cdot h^{2}}$$

where F_flex_—bending force that destroys the sample, S—the span length (100 mm), a—sample width, h—sample height. 

Compressive strength was measured on cube samples using the universal test machine Cometech QC-508 (Taichung, Taiwan). The compressive strength—R_comp_ was calculated using the equation:

(5) $$R_{comp}=\frac{F_{comp}}{a\cdot b}$$

where F_comp_—compressive force that destroys the sample, a—sample length, b—sample width. All measurements were performed on 6 samples. The result was the average of 6 measurements and the standard uncertainty estimated from the standard deviation (the t-Student distribution was used for a significance level of α = 0.05).

## 3. Results

### 3.1. Thermal Characteristics

#### 3.1.1. Determining the Temperature Range for Carbonate Decarbonation

**Figure 2:**
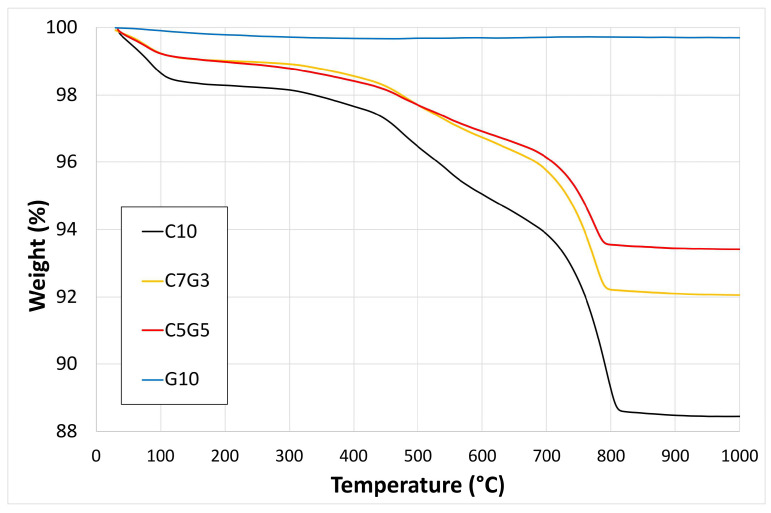
TG curves of raw materials and mixtures.

**Figure 3:**
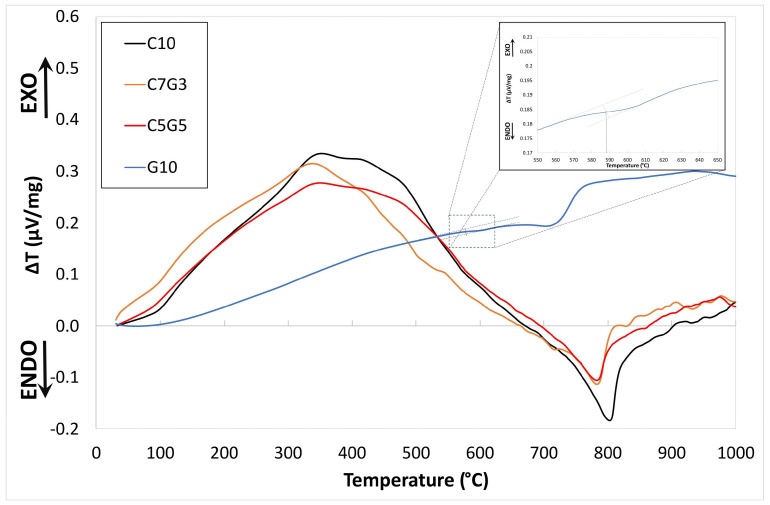
DTA curves of raw materials and mixtures.

**Figure 4:**
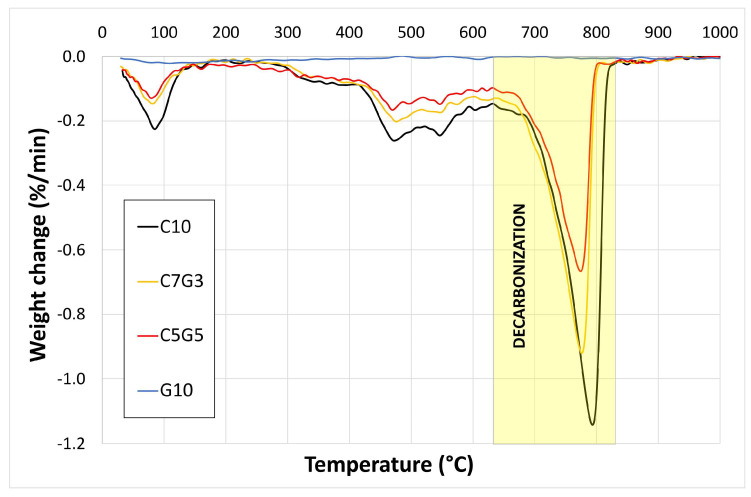
DTG curves of raw materials and mixtures.

**Figure 5:**
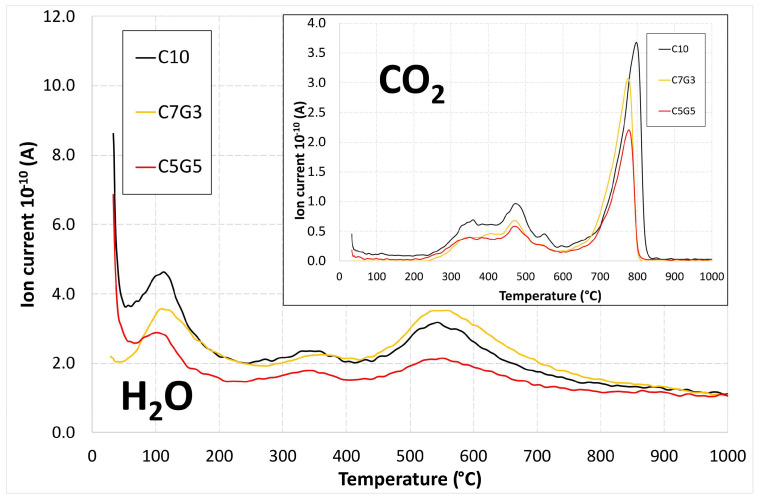
H_2_O (m:18) and CO_2_ (m:44) emission during sintering of raw material and mixtures.

Figure 2, Figure 3, Figure 4 and Figure 5 present the results of thermal measurements for the starting raw materials (clay and glass cullet) and mixtures whose compositions were given in Table 1.

The clay used in the study (C10) is a rock composed of various minerals. During thermal testing, these minerals undergo characteristic thermal transformations. The temperature ranges for the most important thermal effects are listed below:

20–200 °C—endothermic process associated with the emission of H_2_O—dehydration of water physically adsorbed on the surface of all minerals and removal of interstitial water of clay minerals.

250–530 °C—an exothermic process associated with CO_2_ and H_2_O emissions—combustion (oxidation) of organic substances, where initially, at lower temperatures, hydrocarbons are burned (simultaneous CO_2_ and H_2_O emissions) and later only carbon (CO_2_ emissions alone).

500–630 °C—endothermic process associated with H_2_O emission—dehydroxylation of clay minerals.

630–830 °C—endothermic process associated with CO_2_ emissions—decarbonation of carbonates contained in clay.

The glass cullet (G10) used for clay modification undergoes two transformations, which are practically not accompanied by gas emission (negligible mass loss on the TG curve—Figure 2). At a temperature of ~580 °C (enlarged field in Figure 3), the glass transformation occurs, i.e., the pseudo-ordering reaction of the glassy state [36,37]. Above a temperature of 700 °C, the exothermic sintering process begins.

From the research problem point of view, the most important factor was to determine the range of decarbonation temperatures for carbonates contained in the clay raw material. The maximum intensity of this reaction for pure clay is approximately 790 °C (Figure 4). The addition of glass cullet causes a “dilution effect”, a decrease in CO_2_ emission intensity proportional to the amount of introduced (inert) glass additive (Figure 5—internal graph). The addition of glass causes the maximum decarbonation reaction to shift towards lower temperatures. This observation can be combined with the fact that the decarbonation reaction rate depends on the CO_2_ partial pressure. In the pure clay raw material, the amount of CO_2_ released, and therefore the partial concentration of CO_2_ (at a constant gas flow in the DTA/TG apparatus), is the highest among the tested samples; hence, decarbonation shifts towards higher temperatures, or conversely, “dilution” of the clay with glass cullet causes decarbonation to shift towards lower temperatures.

#### 3.1.2. Determining the Firing Temperature—HSM

**Figure 6:**
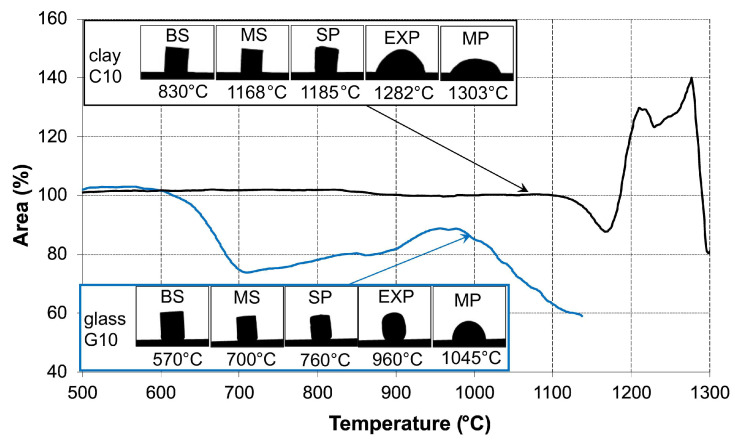
HSM results for raw materials. BS—beginning of sintering, MS—maximum of sintering, SP—the softening point, EXP—maximum of expansion, MP—the melting point.

**Figure 7:**
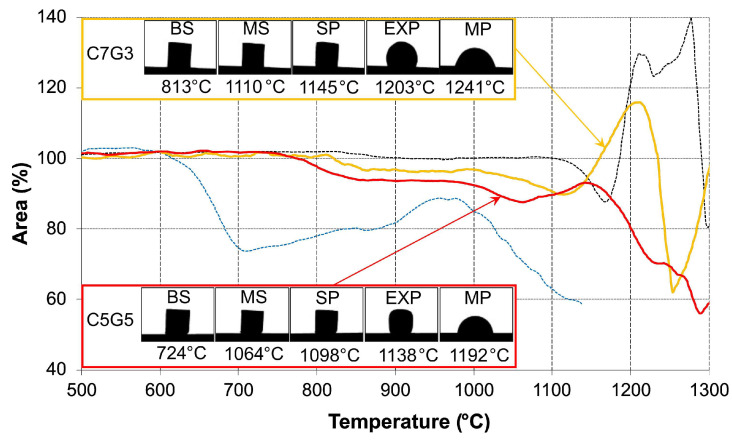
HSM results for ceramic mixtures. BS—beginning of sintering, MS—maximum of sintering, SP—the softening point, EXP—maximum of expansion, MP—the melting point, the dash lines—results for raw materials.

Figure 6 presents the results of HSM measurements obtained for pure raw materials, and Figure 7 presents the results for ceramic mixtures, the composition of which is given in Table 1. These results are presented in the form of graphs of the sample’s shadow surface area as a function of temperature, and in the form of images taken at the so-called characteristic temperatures of the HSM. Table 3 summarizes these temperatures for the raw materials and ceramic mixtures.

Shrinkage is one of the sintering process macroscopic effects and one of the basic parameters for its measurement. However, some initial sintering mechanisms do not cause shrinkage, e.g., free diffusion across grain surfaces and the evaporation–condensation mechanism [38,39]. Sintering shrinkage mechanisms are more pronounced to ensure appropriate performance parameters of ceramic materials such as strength and water absorption. However, a slight increase in mechanical strength is possible through shrinkage-free sintering. The HSM results showed that shrinkage sintering for raw clay material begins at 830 °C. However, at this temperature the calcium carbonate was completely decarbonated. The addition of glass reduces the initial shrinkage sintering temperature even to 724 °C for the C5G5 mass, i.e., to the temperature at which the decarbonation of calcium carbonate is not complete. Thermal tests allowed the determination of the optimal firing temperatures of materials at which calcium carbonate decarbonation is not complete, i.e., 700 °C and 750 °C.

### 3.2. The Influence of Lowering the Firing Temperature on the Microstructure of Ceramic Materials

**Figure 8:**
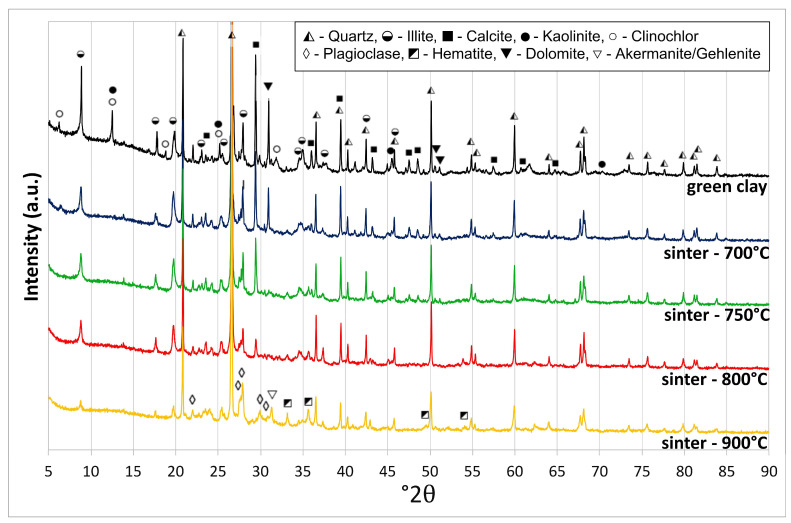
Phase composition of clay raw material and its sinters.

Figure 8 presents the results of phase composition tests for the clay raw material and sinters obtained at different firing temperatures.

The clay contains minerals such as illite (and/or muscovite), kaolinite and clinochlore. It also contains a significant amount of non-clay minerals such as quartz, calcite, dolomite and plagioclase. Thermal treatment of this raw material causes significant changes in the phase composition, but the quantitative description of these changes is limited due to the poor crystallinity of most phases and their very high detection threshold. These minerals include clay minerals (very fine and defective minerals) and plagioclase minerals (solid solutions). Additionally, the high coincidence of the reflections, poor crystallinity of most phases and their generally large number meant that the Rietveld analysis could not be performed correctly. To observe the direction of phase composition changes during thermal treatment, an intensity analysis of the main phase peaks was performed using tools available in the X’Pert HighScore (Plus) program.

Based on this analysis (Table 4), the following conclusions can be drawn. As a result of the dehydroxylation process mentioned in Section 3.1.1, clay minerals become permanently damaged, resulting in the extinction (kaolinite, clinochlore) or a decrease in the intensity of the mineral’s peaks (illite/muscovite). Thermal treatment of calcite (CaCO_3_) and dolomite (CaCO_3_·MgCO_3_) results in their decarbonation. In sinters heated to 900 °C, both carbonates are no longer present. However, what is noteworthy is that solid decarbonation products, primarily free CaO, are undetectable. This means that the CaO is in a non-crystallized (or cryptocrystalline) form and/or, as demonstrated in the chemical studies presented below, the CaO has reacted to form other calcium compounds. The quartz content in sinters changes in a non-directional manner, which is probably related to the polymorphic transformations of SiO_2_ occurring during thermal treatment. The amount of plagioclase also varies in a non-directional manner, with the smallest amount of plagioclase occurring in sinters from a temperature of 800 °C and the largest in sinters from a temperature of 900 °C. Hematite appeared in the sinters, and its amount increased with the firing temperature. The diffraction pattern of the sinter from 900 °C for a diffraction angle of 2θ ≈ 31.3° showed an intense peak, which is difficult to clearly associate with a specific phase. However, calcium disilicates (sorosilicates), whose endmembers are akermanite (hkl 211 plane, 2θ = 31.29°) and gehlenite (hkl 211 plane, 2θ = 31.32°), exhibit intense reflections at this diffraction angle.

**Figure 9:**
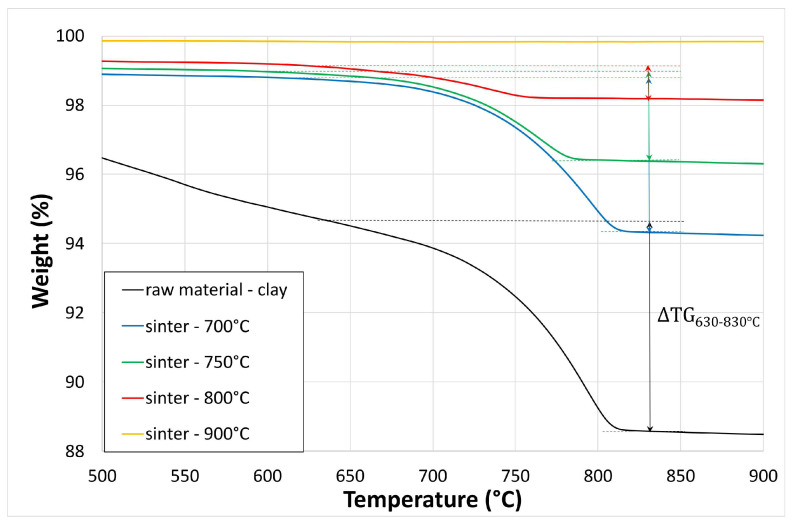
Losses on ignition estimation of sinters and clays in terms of decarbonation. The ignition losses in the decarbonation ranges are marked.

Analysis of the XRD results of the sinters revealed the absence of free CaO. This conclusion led to the verification of this phase presence using the chemical glycol method. The results of these experiments are presented in Table 5. The same sinters and the clay raw material were subjected to thermogravimetric (TG) testing to determine the mass loss during the decarbonation reaction range ∆TG_630–830°C_—Figure 9.

The estimated mass losses due to decarbonation were used to calculate the following:

- the amount calcium carbonate:

∆TG_630–830°C_ · 100/44 (6)

- the amount of CaO bound in calcium carbonate:

∆TG_630–830°C_ · 56/44 (7)

The results of these calculations are presented in Table 5.

**Figure 10:**
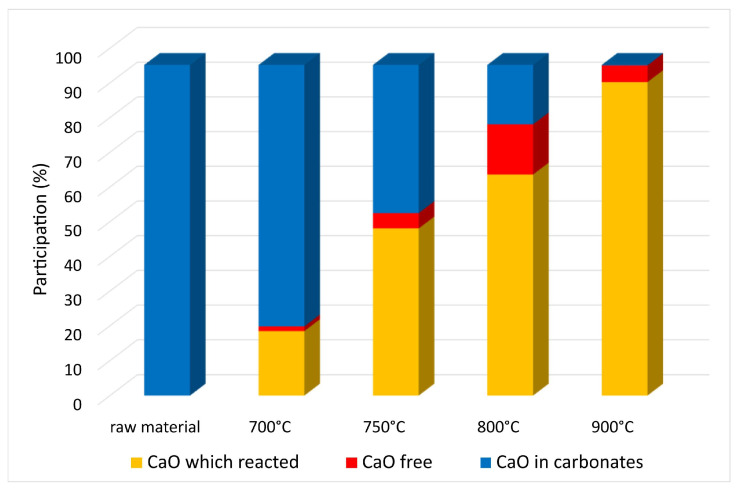
Thermal evolution of calcium compounds in clay raw material.

It should be emphasized that the calculations performed are highly simplified and ignore the presence of dolomite, which results in a higher estimated CaO content in calcium carbonate for the clay raw material than that determined by XRF studies (Table 1). Nevertheless, these estimates allowed for very interesting conclusions, namely, that the CaO formed during carbonate decarbonation quite quickly becomes a substrate for the synthesis of limestone phases. The last row of this table estimates the amount of reacted CaO, which is present neither in calcium carbonate nor as free CaO. Figure 10 illustrates the transformation of calcium compound forms in sinters at different temperatures.

**Figure 11:**
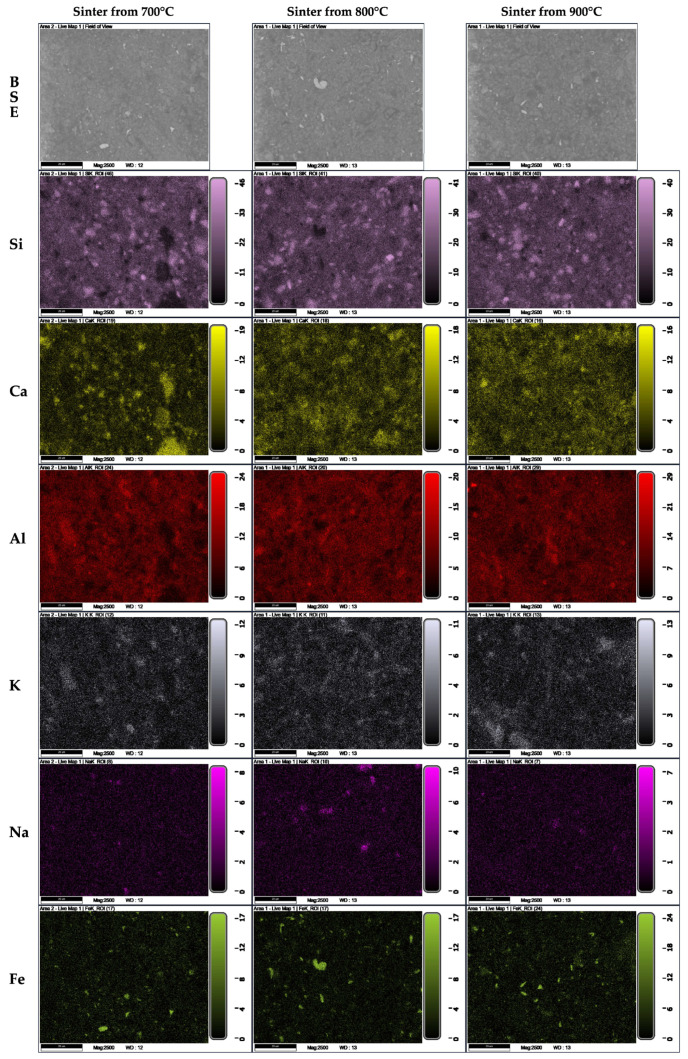
SEM results and element distribution in sinters from different temperatures.

Calculations indicated that calcium compounds in the sinters were synthesized in considerable numbers, but XRD phase studies did not provide an answer as to the specific calcareous phases. Only in the sinters from 900 °C could a larger proportion of plagioclase (sodium–calcium tectosilicates) be detected [40]. The inefficient XRD detection of new calcareous phases probably indicates the cryptocrystalline nature of these phases and/or poor crystallinity due to the formation of solid solutions. The aforementioned plagioclase is an example of such solid solutions. To better understand the nature of the new calcium compounds, the sintered samples from temperatures of 700 °C, 800 °C and 900 °C were subjected to SEM microscopic observations along with EDS mapping, enabling visualization of the spatial distribution of elements. Figure 11 presents the results of these determinations in the form of selected representative areas of individual sinters and maps of the distribution of the main elements—Si, Ca, Al, K, Na and Fe—prepared for these areas.

SEM images of all sinters show a fine-grained microstructure, with most grains less than 5 μm in size. Although the sinters are similar, discrete differences can be observed; however, these differences require prior explanation of the observed microstructural features. Generally, we can assume that three types of microstructural features exist:

- Light, sharp-edged, mostly elongated grains are iron oxides (main elements on EDS: Fe, O).
- Intermediate, sharp-edged grains are dehydroxylated illite/smectite (main elements on EDS: O, Si, Al, K), quartz (Si, O), calcium silicates (O, Si, Ca), magnesium and calcium aluminosilicates (O, Si, Mg, Ca, Al) and calcium oxide (Ca, O).
- Dark areas are the matrix of lighter grains—the filling phase is calcium aluminosilicates (O, Si, Ca, Al).

The observed microstructures differ in the amount and thickness of the last, darker filling phase. In the sinter from 900 °C, the amount and thickness of this phase are greatest. It is probably not a single type of phase, but it is likely largely formed by synthesis products. Analysis of elemental distributions is also similar. Aside from the distribution of Fe (which is concentrated in iron oxides), there are no significant contrasts in the distribution of the other elements. This is a result of fine-grainedness and high defects in the phases (e.g., dehyroxylated clay minerals), as well as the presence of solid solutions with a wide miscibility range (e.g., plagioclase). The only element for which relatively objective differences in the distribution between individual sinters can be observed is Ca. The higher the thermal treatment temperature, the less concentrated this element is in the areas.

**Figure 12:**
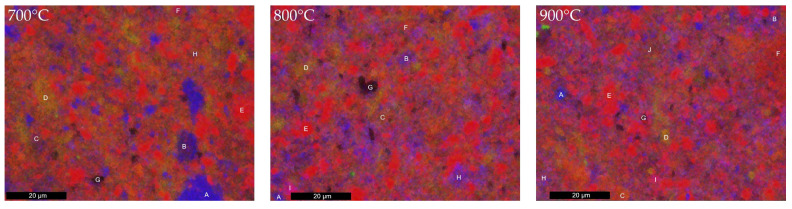
SEM images with marked areas characteristic for sinters from different temperatures based on the overlay of element maps: Ca (blue), Al (green), Si (red).

**Figure 13:**
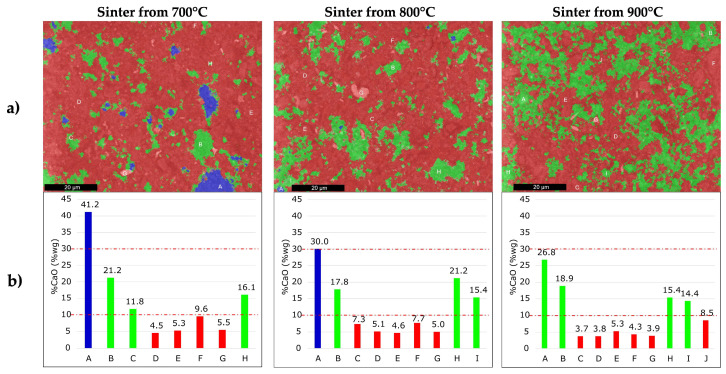
Distribution of CaO in sinters from different temperatures based on elemental map analysis: blue wt%CaO ≥ 30%, green wt%CaO 10–30%, red wt%CaO < 10%. (**a**) CaO content distribution maps, (**b**) CaO content in separate areas.

Figure 12 presents a summary of SEM images resulting from the analysis of elemental maps, along with the marking of characteristic areas. Table 6 summarizes the estimated oxide composition of characteristic areas. In Figure 13, to highlight the difference in Ca distribution, the areas of this element content (converted to CaO weight percentage) were also overlaid on the BSE images, along with the addition of bar graphs of the content for characteristic areas. Blue indicates areas with CaO weight percentage greater than or equal to 30%, green indicates areas in the range of 10–30%, and red indicates areas below 10%.

The analysis presented in Figure 12 and Figure 13 and Table 6 confirms the earlier conclusion that Ca is more dispersed in sinters from higher firing temperatures. Therefore, it is a substrate that readily diffuses, simultaneously forming calcium compounds. XRD analysis could not unambiguously determine which new calcium compounds were formed, but it was known that they must be formed, as free CaO resulting from the decomposition of calcium carbonate was barely detectable in ceramic sinters using the glycol method (Figure 10). EDS analysis of the points indicates that these new components are likely primarily calcium aluminosilicates (Ca-rich plagioclase), but calcium silicates such as calcium orthosilicate and/or rankinite cannot be ruled out.

### 3.3. Technological Research

Table 7 presents the basic parameters of ceramic sinters obtained at different temperatures, where the pure raw material was fired at temperatures of 700 °C, 750 °C, 800 °C and 900 °C, while the mixtures with the addition of glass were tested only in the range of incomplete decarbonation of calcium carbonate—700 °C and 750 °C.

During the molding stage, it was observed that the mixes with the addition of cullet exhibited lower plasticity, meaning the cullet addition acts as a thinner during the molding stage. Furthermore, the higher the cullet addition to the molding mix, the lower the vacuum generated by the deaeration pump. These observations indicate that the addition of glass increases gas permeability through the molding mix, likely by reducing grain packing. Despite this effect of the glass addition to the molding mixes, the C7G3 mix continued to demonstrate good molding properties, while the C5G5 mix exhibited molding defects (torn edges of the emerging strand), indicating an overdose of the additive.

Materials obtained from pure clay raw materials at temperatures of 700–800 °C exhibit negative sintering shrinkage (expansion). Samples with the addition of cullet from a temperature of 700 °C also experience expansion, but to a lesser extent. The less clay raw material and calcium carbonate content, the lower the expansion during firing. This should therefore be attributed to the intense CO_2_ release resulting from the decomposition of calcium carbonate, which is confirmed by literature reports [41]. In the C5G5 sample fired at 750 °C, a sintering shrinkage of about 1% was recorded, while in the 900 °C sintered pure clay, the sintering process compensated for the above-mentioned expansion caused by CO_2_ emissions. The bulk density of beans obtained from pure clay is the highest in the sinter from a temperature of 700 °C, at higher temperatures the density gradually decreases with the progress of calcium carbonate decarbonation, reaching a minimum in the sinter from a temperature of 800 °C. At higher firing temperatures (900 °C), the bulk density of the sinter increases, which is attributed to sintering shrinkage. All sinters obtained from the raw material mixture (clay + cullet) have lower bulk densities compared to sinters obtained from pure clay. This observation is attributed to a lower packing degree of the mixtures during molding and after drying. The densities of the shapes after drying (before firing) were as follows: C10—1937 ± 5 kg/m^3^, C7G3—1806 ± 9 kg/m^3^, C5G5—1725 ± 5 kg/m^3^. Water absorption is a parameter directly proportional to the open porosity of the materials. For materials obtained from pure clays, water adsorption initially increases, which is attributed to the progressive decomposition of calcium carbonate. In sinters obtained from a temperature of 800 °C, it reaches a maximum value of 17.5% and then decreases due to sintering, which causes the mass to densify. The same processes (decarbonation and sintering) affect the final water absorption of sinters obtained from mixtures of clay and glass cullet; however, their final water absorption is similar at ~16%. The results for the compressive and flexural strength of the materials are notable. Not surprisingly, both strengths increase with increasing thermal treatment temperature. However, the effect of the addition of glass cullet on these parameters is peculiar. This additive significantly improves flexural strength but has virtually no effect on compressive strength, and may even reduce strength, as in the case of the C5G5 sample fired at 700 °C. Glass cullet undoubtedly accelerates sintering processes (including non-shrinkage sintering), but it causes a lower packing degree of the beans during forming, thus reducing bulk density and increasing the overall porosity of the materials. The flexural strength of materials obtained from clay and cullet mixtures was less affected (reduced) by their lower packing density, but was strongly affected (increased) by the sintering processes initiated by the addition of cullet. It is likely that the intensification of sintering induced by the addition of glass significantly reduced the size of the so-called critical crack propagation gap [38]. Compressive strength, however, is primarily related to the porosity of the materials. In this case, only shrinkage sintering mechanisms can significantly increase compressive strength, as they reduce the porosity of the sintered materials. Non-shrinkage sintering mechanisms do not reduce porosity, and compressive strength can be slightly increased due to the interconnections between the grains. A final interesting finding concerns ceramic materials obtained from pure clays. As can be seen, the compressive strength of sinters from temperatures of 750 °C and 800 °C is practically the same. The cause of this “anomaly” is the balance of the two factors discussed above that have an antagonistic effect on compressive strength: increased porosity due to calcium carbonate decarbonation and grain consolidation due to sintering. Therefore, from the perspective of improving the compressive strength of ceramic materials, increasing the thermal treatment temperature is not always justified.

### 3.4. Summary—Reducing CO_2_ Emissions

In accordance with the aim of the work (reduction of CO_2_ emissions through incomplete decomposition of calcium carbonates) and based on the results obtained in Section 3.3, the following summary points were established:

1. In the case of the production of masonry ceramics (from clays containing carbonates), where the most important mechanical parameter is compressive strength, the optimal firing temperature is 750 °C. At this temperature, approximately 45% of the carbonates remain undecomposed (5.7% remaining). Taking into account the molar masses of calcium carbonate (100) and CO_2_ (44), and assuming that the carbonates occur only in the form of calcium carbonate, the percentage of CO_2_ in the undecomposed carbonate can be calculated:

(8) $$\%{\mathrm{CO}}_{2}\;=\;5.7\%\frac{M_{CO2}}{M_{CaCO3}}\approx \;2.50\%$$

The process emission intensity is EM_process_ = 25.0 kg (CO_2_/ton of clay).

Furthermore, the heat required to decompose calcium carbonate is increase, further increasing fuel emissions. This CO_2_ extra emission can be calculated as follows:

- Calculate the number of undecomposed carbonate moles (5.7%—57 kg) in one ton of raw material:

n = m_CaCO3_/M_CaCO3_ (570 moles of CaCO_3_) (9)

- Calculate the energy E needed to decompose this number of moles of carbonate. The heat of decomposition of calcium carbonate C = 178 kJ/mole:

E = n·C = (101.5 MJ = 0.1015 GJ)(10)

- Assuming typical natural gas emissions EM_gas_ = 56 kg CO_2_/GJ, the amount of additional fuel emissions is:

EM_fuel_ = E·EM_gas_ (5.68 kg CO_2_/ton of clay) (11)

The total reduction in CO_2_ emissions when reducing the firing temperature to 750 °C is over 30 kg for every 1 ton of clay raw material used. Materials obtained from pure clay, fired at 750 °C, achieve parameters typical of high-strength wall ceramics, for example, 25 MPa according to EN 771-1:2011+A1:2015. Porous additives can still be added to clay to obtain porous products for masonry applications. [42,43]. The addition of glass cullet does not provide measurable results in improving compressive strength, but it does reduce the proportion of clay raw material in the mass, which reduces the mass’s emission capacity. However, the level of CO_2_ emission reduction is difficult to estimate because, as can be seen in Figure 4 and Figure 5, it is not proportional to the level of clay substitution with glass cullet. Any potential applications of the concept of lowering the firing temperature to 750 °C must be preceded by technological research that considers the durability of the materials, including resistance to changing humidity conditions and dimensional stability. Such research may limit the products’ application, for example, only to enclosed walls.

2. The addition of cullet is beneficial for products that need to compensate for the decrease in flexural strength associated with lower firing temperatures. However, due to the deterioration of forming properties and the decrease in compressive strength (caused by lower packing after forming), the cullet content in the mass cannot exceed 30% by weight.

## 4. Conclusions

In this paper, research aimed at reducing CO_2_ emissions from decarbonation during the firing of construction ceramics made from clays containing calcium carbonate was conducted. This goal was achieved by lowering the firing temperature of these materials and using an intensive waste flux (glass cullet). Knowing that such radical technological procedures cause specific microstructural effects, their analysis was an intermediate goal. Based on the research, the following conclusions were drawn:

1. The temperature range for decarbonation of calcium carbonate in clay and clay–cullet mixtures was 630–830 °C, while shrinkage sintering in clay begins at 830 °C. The addition of glass cullet reduced the shrinkage sintering temperature by more than 100 °C in the case of the C5G5 mixture.
2. Calcium carbonate decarbonation occurring in fired materials hinders sintering, causing slight expansion and increased open porosity. These changes are more pronounced the higher the carbonate content in the sintered raw material.
3. The free calcium oxide formed during sintering reacts quite quickly to form calcium aluminosilicates (plagioclase) and probably also calcium silicates such as calcium orthosilicate and/or rankinite.
4. The high kinetics of the calcium compound synthesis reaction is probably dictated by two factors: the high calcium diffusion coefficient (Figure 13) and the high activity of dehydroxylated clay minerals.
5. The addition of glass cullet to clay containing calcium carbonate is beneficial in terms of improving the flexural strength of the materials, but does not bring any benefits in terms of the compressive strength.
6. The lack of a beneficial effect, or negative effect occurrence, of adding glass cullet to ceramic materials on their compressive strength after firing is caused by a lower degree of grain packing of ceramic masses already at the forming stage.
7. To achieve the main goal of this paper (reducing CO_2_ emissions from calcium carbonate decarbonation), it is best to sinter materials in the temperature range of 700–750 °C. At these temperatures, non-shrinkage sintering mechanisms dominate, such as diffusion along the grain surface and diffusion through the gas phase.

## Figures and Tables

**Table 1:** Designation and composition of ceramic mixtures.

Designation	Content (% *w*/*w*)	
	Clay Raw Material	Glass Cullet
C10	100	0
C7G3	70	30
C5G5	50	50
G10	0	100

**Table 2:** Chemical composition of the clay and the glass.

Sample	Component Content (wt%)									
	SiO_2_	Al_2_O_3_	Fe_2_O_3_	CaO	MgO	SO_3_	Na_2_O	K_2_O	Rest	LOI
Clay	53.65	14.57	4.82	6.83	3.05	0.10	1.13	2.74	1.65	11.46
Glass	67.34	2.39	0.46	10.17	1.01	0.08	15.58	0.44	2.24	0.29

**Table 3:** HSM results for raw materials and ceramic mixtures.

Designation	Characteristic Temperature (°C)				
	Beginning of Sintering	Maximum of Sintering	The Softening Point	Maximum of Expansion	The Melting Point
C10	830	1168	1185	1282	1303
C7G3	813	1110	1145	1203	1241
C5G5	724	1064	1098	1138	1192
G10	570	700	760	960	1045

**Table 4:** Characteristics of the selected phase peak intensity occurring in the raw material and sinters.

Phase:	h k l	2θ (°)	Peak Area (cts·2θ)				
			Raw	700 °C	750 °C	800 °C	900 °C
Quartz	0 1 1	26.64	3232	1864	2360	2464	3340
	1 1 2	50.15	366	211	220	250	324
Illite/Muscovite	0 0 2	8.86	357	164	100	107	62
Calcite	1 0 4	29.41	710	368	259	68	-
Kaolinite	0 0 1	12.51	127	-	-	-	-
Clinochlor	0 0 1	6.26	38	18	-	-	-
Plagioclase	0 4 0	27.97	265	192	173	123	288
Hematite	1 0 4	33.17	-	18	25	30	45
Dolomite	1 0 4	30.95	352	137	22	-	-

**Table 5:** Forms of CaO occurrence in sinters from different temperatures—calculation results.

Parameter:	Raw	700 °C	750 °C	800 °C	900 °C
Content CaO _free_—glycol method (%)	0	0.10	0.33	1.08	0.36
∆TG—from 630 °C to 830 °C (%)	5.87	4.47	2.57	1.01	0.004
Content CaCO_3_ (%)	12.70	10.06	5.70	2.13	0.005
Content CaO in CaCO_3_ (%)	7.11	5.63	3.19	1.28	0.01
Content CaO which reacted (%)	0.00	1.38	3.59	4.75	6.74

**Table 6:** Oxide composition of characteristic areas estimated on the elemental maps analysis.

Area	Temperature (°C)	Component Content (wt%)							
		SiO_2_	Al_2_O_3_	CaO	Fe_2_O_3_	SO_3_	K_2_O	MgO	Na_2_O
A	700	33.3	9.0	41.2	6.7	2.9	2.3	4.2	0.3
	800	38.3	14.0	30.0	6.1	5.1	3.1	3.2	0.2
	900	26.7	14.6	26.8	24.8	1.7	2.4	2.8	0.2
B	700	40.2	13.5	21.2	6.4	2.8	1.8	13.9	0.3
	800	42.7	13.1	17.8	9.2	2.0	2.2	12.9	0.2
	900	44.8	15.3	18.9	8.2	7.4	3.0	2.3	0.2
C	700	47.0	17.7	11.8	12.0	2.9	3.6	4.6	0.3
	800	53.5	19.4	7.3	11.2	1.2	3.3	3.8	0.2
	900	61.5	20.0	3.7	5.9	1.2	6.3	1.2	0.2
D	700	54.6	25.6	4.5	4.7	1.6	4.8	3.9	0.2
	800	52.4	25.8	5.1	7.5	1.2	4.9	3.0	0.2
	900	57.6	26.7	3.8	3.4	0.5	1.9	5.9	0.2
E	700	75.3	8.5	5.3	3.6	2.3	2.0	2.9	0.2
	800	77.0	8.3	4.6	5.0	1.2	2.2	1.6	0.1
	900	76.7	9.9	5.3	3.4	0.6	1.8	2.1	0.2
F	700	57.0	11.9	9.6	9.7	1.7	2.3	7.5	0.3
	800	60.9	16.1	7.7	6.9	1.2	4.8	2.2	0.3
	900	56.5	13.5	4.3	14.7	1.2	5.8	3.7	0.3
G	700	23.9	8.0	5.5	55.7	2.4	2.1	2.0	0.4
	800	22.7	5.9	5.0	61.3	1.2	2.1	1.6	0.1
	900	28.3	8.5	3.9	53.8	1.4	1.5	2.3	0.4
H	700	41.6	17.3	16.1	8.6	11.3	2.5	2.3	0.1
	800	47.1	14.2	21.2	8.9	2.8	2.6	3.0	0.2
	900	53.1	19.3	15.4	4.2	2.3	1.7	3.9	0.2
I	700	N/A	N/A	N/A	N/A	N/A	N/A	N/A	N/A
	800	63.3	10.1	15.4	6.0	1.4	2.2	1.5	0.1
	900	63.4	9.9	14.4	8.0	0.7	1.7	1.8	0.2
J	700	N/A	N/A	N/A	N/A	N/A	N/A	N/A	N/A
	800	N/A	N/A	N/A	N/A	N/A	N/A	N/A	N/A
	900	53.5	16.7	8.5	10.7	0.6	5.0	4.8	0.3

**Table 7:** Physical parameters of ceramic materials.

	Properties	Firing Shrinkage (%)	Bulk Density (kg/m^3^)	Water Absorption (%)	Flexural Strength (MPa)	Compressive Strength (MPa)
Sinter						
C10	700 °C	−0.39 ± 0.05	1808 ± 6	15.1 ± 0.1	7.5 ± 1.1	26.7 ± 1.0
	750 °C	−0.44 ± 0.04	1769 ± 5	15.8 ± 0.1	9.8 ± 0.9	32.5 ± 2.6
	800 °C	−0.33 ± 0.05	1743 ± 8	17.5 ± 0.2	11.5 ± 1.2	32.2 ± 2.3
	900 °C	0.04 ± 0.06	1764 ± 8	16.7 ± 0.2	13.2 ± 1.0	42.5 ± 1.9
C7G3	700 °C	−0.28 ± 0.05	1712 ± 6	15.6 ± 0.2	11.6 ± 1.1	25.0 ± 1.5
	750 °C	−0.12 ± 0.06	1708 ± 3	16.0 ± 0.2	15.0 ± 0.9	32.2 ± 2.0
C5G5	700 °C	−0.13 ± 0.04	1660 ± 6	16.6 ± 0.2	12.2 ± 0.7	23.8 ± 1.7
	750 °C	0.92 ± 0.13	1700 ± 12	15.4 ± 0.3	18.5 ± 0.7	32.3 ± 2.6

## Data Availability

The raw data supporting the conclusions of this article will be made available by the authors on request.
